# Estrogen and progesterone attenuate glutamate neurotoxicity via regulation of EAAT3 and GLT-1 in a rat model of ischemic stroke

**DOI:** 10.22038/ijbms.2020.48090.11039

**Published:** 2020-10

**Authors:** Sara Nematipour, Zeinab Vahidinia, Majid Nejati, Homayon Naderian, Cordian Beyer, Abolfazl Azami Tameh

**Affiliations:** 1 Anatomical Sciences Research Center, Institute for Basic Sciences,Kashan University of Medical Sciences, Kashan, Iran; 2 Department of Anatomy, School of Medicine, Iran University of Medical Sciences, Tehran, Iran; 3 Institute of Neuroanatomy, Faculty of Medicine, RWTH Aachen University, Aachen, Germany

**Keywords:** EAAT3, Estrogen, GLT-1, Glutamate transporter, Progesterone, tMCAO

## Abstract

**Objective(s):**

Glutamate is the most widespread neurotransmitter in the central nervous system and has several functions as a neuromodulator in the brain although in pathological conditions like ischemia it is excessively released causing cell death. Under physiological conditions, glutamate is rapidly scavenged from the synaptic cleft by excitatory amino-acid transporters (EAATs). An imbalance in glutamatergic neurotransmission could influence the expression of glutamate transporters and is a pathological feature in several neurological disorders. It has been shown that estrogen and progesterone act as neuroprotective agents after brain injury. This study aims to investigate the role of hormone therapy after middle cerebral artery occlusion (tMCAO) in the expression of GLT-1 and EAAT3 as glutamate transporters.

**Materials and Methods:**

Middle cerebral artery occlusion technique was performed in Wistar rats in order to induce focal cerebral ischemia. Estrogen, progesterone, and a combination of both hormones were injected subcutaneously in the early minutes of reperfusion. Sensorimotor functional tests were performed and infarct volume was calculated by TTC staining of brain section. Gene and protein expression of EAAT3 and GLT-1 were evaluated by RT-PCR, immunoblotting, and immunohistochemistry.

**Results:**

Behavioral scores were increased and infarct volume was reduced by hormone therapy. RT-PCR, immunoblotting, and immunohistochemistry data showed that the expression of GLT-1 and EAAT3 increased after ischemia. Also, estrogen and progesterone treatment enhanced mRNA and protein expression levels of GLT-1 and EAAT3 compared with ischemia.

**Conclusion:**

Steroids may protect brain tissue against ischemia-induced tissue degeneration by decreasing extracellular glutamate levels through the induction of glutamate transporters.

## Introduction

Stroke is the major cause of long-term disability and the third most common reason for mortality after cardiovascular disease and cancer worldwide (1, 2). Focal ischemia is a major type of stroke that results from an embolic occlusion of the middle cerebral artery (MCA) and causes neuronal damage in the cerebral cortex and striatum (3, 4). Neural death after ischemia happens due to several events including oxidative stress, inflammation, increased intracellular calcium levels, glutamate-induced excitotoxicity, apoptosis, and necrosis (5-8). Glutamate is a neurotransmitter that is involved in many physiological functions of the brain (9). However, excess glutamate secretion in the extracellular space can cause neurotoxicity. This phenomenon leads to the excitotoxicity and death of both neurons and glial cells (2, 10). Under physiological conditions, glutamate is rapidly removed from the synaptic cleft by excitatory amino acid transporters (EAATs) which represents a vital process for neuronal survival (11, 12). EAATs are found in astrocytes and neurons. EAAT1 (GLAST) and EAAT2 (GLT-1) are expressed on astrocytes while EAAT3 (EAAC1), EAAT4, and EAAT5 are expressed in neurons (13-15). Under pathological conditions like brain ischemia, a massive overload of glutamate release causes an over-activation of glutamate receptors such as N-methyl-D-aspartate receptors (NMDA). This process increases the concentration of Ca^++^ in the intracellular space which leads to necrosis or apoptosis (16, 17). In addition, disturbance of glutamate neurotransmission due to changes in glutamate transporters can be considered a cause for some neurological diseases, including ischemia and multiple sclerosis (MS) (15, 18). In the penumbra region which surrounds the ischemic core neurons are still alive and have the potential to be saved through post-ischemic therapies. Thus, one of the primary goals of experimental therapeutic approaches is the protection and rescue of neurons in this region (19, 20). 

The risk of neurodegenerative diseases in postmenopausal women is higher compared with young women (21, 22). This observation prompted research to consider sex steroids as therapeutic molecules in stroke, Alzheimer’s disease, MS, and amyotrophic lateral sclerosis (18, 23, 24). Estrogen (E2) and progesterone (P) appear to be neuroprotective under experimental stroke-like conditions (25, 26), including transient middle cerebral artery occlusion (tMCAO) (27-29). Although E2 and P-mediated neuroprotection are well-described, the mechanism of this phenomenon is not fully understood. Some evidence indicates that E2 may exert its neuroprotective effects via the excitatory neurotransmitter systems, specifically glutamate receptors and transporters (30, 31). Previous studies showed that steroid precursors like cholesterol increase glutamate transporter clusters on the cell membrane (32). This implicates that sex steroids facilitate the re-uptake of glutamate and thereby protect neurons (33). Here, we focused on alteration of GLT-1 and EAAT3 expression as glutamate transporters in the rat brain after tMCAO and the possible effects of steroid hormones on such changes.

## Materials and Methods

***Animals***

Experiments were performed using adult male Wistar rats (230–270 g). Animals were retained in a pathogen-free and temperature-controlled environment with free access to food and water. All experimental protocols were approved by the Research Ethical Committee, Kashan University of Medical Sciences and were carried out in accordance with Directive 2010/63/EU on the protection of animals used for scientific purposes. Rats were randomly divided into five groups: (1) sham, (2) ischemia/reperfusion (I/R), (3) E2/P-treated and I/R (I/R-E2/P), (4) E2-treated and I/R (I/R-E2), and (5) P-treated and I/R (I/R-P). There was a single dose of E2 (25 µg/kg), P (10 mg/kg) or E2/P (25 µg/kg and 10 mg/kg respectively). Totally 30 rats were subjects in this study. The behavioral exam was evaluated in all groups (N= 6). Fresh brain tissue of 15 rats was used for infarct volume measurement, Western blot, and real-time PCR in all 5 groups (N=3). IHC was performed on fixed brain tissue of rats in 3 groups (1, 2, and 3, N=5).

***Middle cerebral artery occlusion*** ***and hormone treatment***

Artery occlusion was induced by intraluminal tMCAO as previously described with some minor modifications (34). Briefly, animals were anesthetized under 3% isoflurane (Baxter, USA) using isoflurane vaporizer (Eickemeyer, Germany). Body temperature was maintained throughout operation at 37±0.5 °C via a heating pad (NARCO Bio-systems, USA). Laser Doppler flowmetry (Moor Instruments, England) was applied to monitor cerebral blood flow (CBF) during surgery and detection of subarachnoid hemorrhage. Laser Doppler optical probes (P10d) were placed over the thinned skull (using a high-speed dental drill) on the left and right parietal bones (1–2 mm posterior and 4–5 mm lateral to the bregma). After a midline cervical skin incision, the left common carotid artery (CCA) was carefully dissected from the vagus nerve. A silicon-coated monofilament (Doccol, USA) was inserted through CCA into the internal carotid artery (ICA) until a significant drop in CBF (more than 60%) was seen. After 1 hr, the catheter was removed to allow the reperfusion of MCA territory. Rats in the sham group underwent similar operations except for the insertion of a catheter in MCA. For hormone treatment, the following steroid concentrations were used: 25 µg/kg E2 and 10 mg/kg P (Sigma–Aldrich, Germany, initially diluted in pure ethanol) were given as neck depots in 500 µl sesame oil immediately after MCAO induction. Control animals received an appropriate amount of ethanol/sesame oil mixture (29, 35).

***Neurological assessment***

Neurobehavioral tests were scored 24 hr after I/R by an *examiner who *was* blinded *to the experimental protocol as previously described in detail with minor modifications (36). Six sensorimotor tests (spontaneous activity, symmetry in the movement of four limbs, forepaw outstretching, climbing, body proprioception, and response to vibrissae touch) were done to evaluate neurological damage. Individual scores of all tests were summed up at the end of the assessment (maximum and minimum scores were achievable 18 and 3, respectively).

***Infarct volume measurement***

To determine the infarct volume, the rat’s brains were removed and placed in the brain matrix (Zivic Instruments, USA) 24 hr post-tMCAO. Coronal slices with 2 mm thickness were shaken in a 1% 2,3,5-triphenyl tetrazolium chloride (TTC) solution in PBS for 20 min at 37 ^°^C. Brain white matter and infarct tissues remained white, whereas normal active tissues were typically stained red. After the TTC staining, all slices were arranged in order from rostral to caudal, and digital images were taken using a digital camera (Nikon, Japan). The infarct area was assessed using the Image J software package (version 1.44p, USA). Infracted areas of all sections were measured (mm^2^), data summed, and then multiplied by the distance between the sections (2 mm) in order to get the total infarction volume (37).

***Immunoblotting***

Tissue samples were dissected from the penumbra region of TTC-stained sections, put in liquid nitrogen, and stored at -80 ^°^C until later use. For protein extraction, samples were homogenized using Radio-immunoprecipitation Assay buffer (RIPA) containing a cocktail of protease inhibitors. Homogenate samples were centrifuged at 14,000 rpm for 20 min at 4 °C and supernatants were prepared in 2× Laemmli sample buffer and normalized in an equal amount of volume and protein content. Samples were loaded on a 10% sodium dodecyl sulfate-polyacrylamide mini-gel (SDS) electrophoresis. After separation, proteins were blotted onto a PVDF membrane (Roche, Germany) and blocked for 1 hr with 5% skim milk. PVDF membranes were incubated with anti-GLT-1 (1:1,000, Abcam, Germany) as the first antibody. After washing, the blots were incubated for 60 min at room temperature with HRP-labeled secondary antibody (1:3,000, Abcam, Germany) and were visualized by the ECL detection Kit (Peqlab VWR, Germany). Protein band density was measured by ImageJ software. Densities of GLT-1 bands as target protein were normalized to β-actin bands from the same samples as an internal control.

***RNA isolation and real-time PCR***

Total ribonucleic acid (RNA) of samples was extracted using Accuzol reagent (Bioneer, Korea) according to the manufacturer’s instructions. Total RNA was subsequently transcribed into cDNA using iScript™ Reverse Transcription Supermix (BioRad, Germany). After cDNA synthesis, all samples were diluted in 1:10 ratio. Real-time RT-PCR reaction was performed in a reaction mixture consisting of 5 μl SYBR green master mix (BioRad, Germany), 2 μl water, 0.5 μl of each primer (10 μM stock), and 2 μl of diluted cDNA by IQ5 system (BioRad, Puchheim, Germany). Relative standard curve method was used as previously described (15).

Primers were designed using Primer 3 software, matched with the NCBI database using BLASTN, and then optimum annealing temperature was determined by gradient PCR. Results were calculated with the ratio of target gene quantity value to reference gene hypoxanthine-phosphoribosyl transferase (HPRT) value.

The sequences of forward (F) and reverse (R) primers were: GLT-1 F: 5ʹCTGCCCGTTAAATACCGCTC3ʹ, R:5ʹGTCAGTGAGAGCAGGAGGTT3ʹ; EAAT3 F: 5ʹCCTGGTCC

AAGCCTGTTTTC3ʹ, R: 5ʹTGAGTACAGGCCCACGATTT3ʹ; HPRTF: 5ʹGCTCGAGATGTCATCAAGGAGA3ʹ, and R: 5ʹTCAGCGCTTTAATGTAATCCAGC3ʹ. 

***Immunohistochemistry***

Animals were deeply anesthetized and perfused transcardially by normal saline and then by 10% Neutral-buffered Formalin (NBF, pH= 7). Fixed brains were carefully removed, cut into 4 mm thick slices, and kept in the same fixative for 48 hr at room temperature. After tissue processing and paraffin-embedding, 5 μm thick coronal brain sections were made using a microtome (Diapath, Italy). Then, sections were deparaffinized and rehydrated, and the antigens were retrieved by heated 0.1 M citrate buffer (pH= 6) for 20 min. Non-specific antibody binding was blocked with a superblock solution from the Immunohistochemistry kit (Scytek, USA) for 7 min at room temperature. Then slices were incubated with rabbit monoclonal EAAT3 antibody (1:1000, Abcam, Germany) overnight at 4 °C. After washing, sections were incubated in 10% H2O2 in methanol (10 min) to prevent reaction with endogenous peroxidases. Then, sections were treated with a biotin-conjugated secondary antibody followed by streptavidin-peroxidase subsequently for 10 min. The color reaction was developed with diaminobenzidine (DAB). For negative control*, *slides underwent identical preparation except that the primary antibody was omitted. The sections were analyzed with a Nikon microscope (Nikon Eclipse Ti-U, Japan).

***Statistical analysis***

Data were analyzed using SPSS software (ver. 22.0, USA) and expressed as means±SEM. For comparison, the data related to CBF repeated measurement ANOVA was used. The behavioral data were analyzed by Kruskal–Wallis test. All other comparisons were performed by one-way ANOVA and *post hoc* Tukey’s test. A probability of *P*≤0.05 was considered significant.

## Results

***CBF course and neurological function***

**Figure 1:**
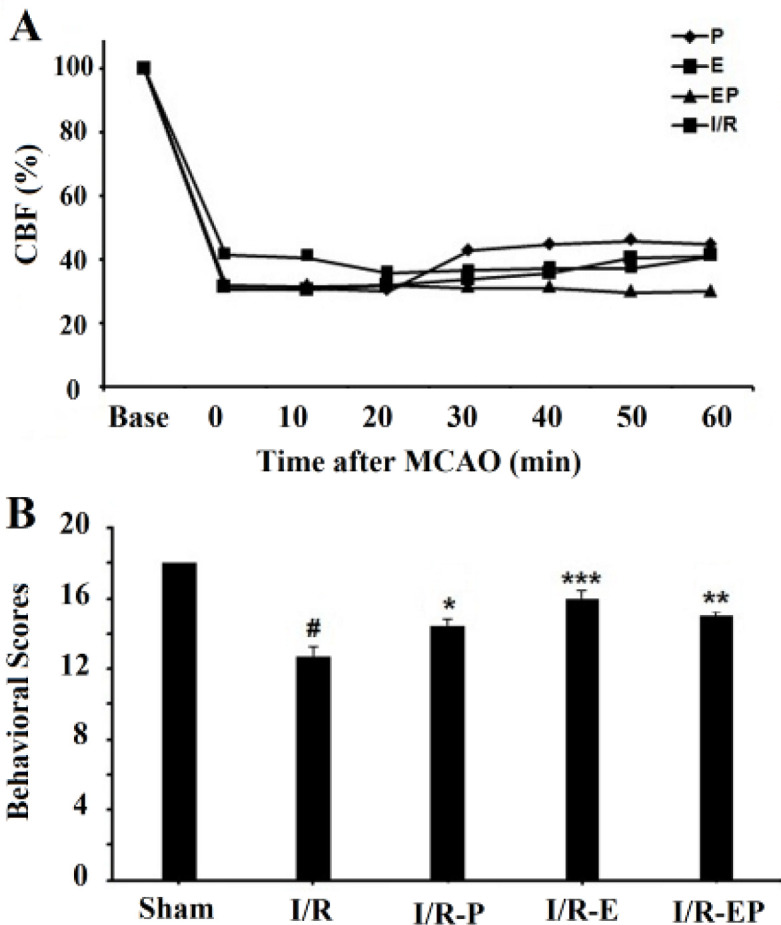
Laser-Doppler measurement of relative cerebral blood flow (rCBF) over the ipsilateral MCA territory before and during 60 min tMCAO (A). The behavioral test showed that the scores of sensory and motor exams significantly decreased 24 hr after I/R compared with the sham group. Estrogen and progesterone treatment in E2, P, and E2/P groups significantly increased behavioral scores compared with the I/R group (B). (*P≤0.05, **P≤0.01, ***P≤0.001, #*P*≤0.05)

CBF: cerebral blood flow; MCA: middle cerebral artery; tMCAO: transient middle cerebral artery occlusion; I/R: ischemia/reperfusion

Laser-Doppler monitoring revealed that all rats included in the experiment had regional blood flow reductions of ≥ 60% immediately after induction of tMCAO (Figure 1A) which remained stable during 1 hr tMCAO. There was no significant difference in the CBF values between different groups. Behavioral scoring dropped significantly in the I/R group compared with the sham group (*P*≤0.05) 24 hr after tMCAO (Figure 1B). Hormone therapy significantly improved neurological function, and behavioral scores elevated from 12.72 in the I/R group to 14.6 in P-treated (*P*≤0.05), 16 in E2- treated (*P*≤0.001), and 15 in E2/P- treated groups* (P*≤0.01) (Figure 1B).

***Infarct volume***

**Figure 2:**
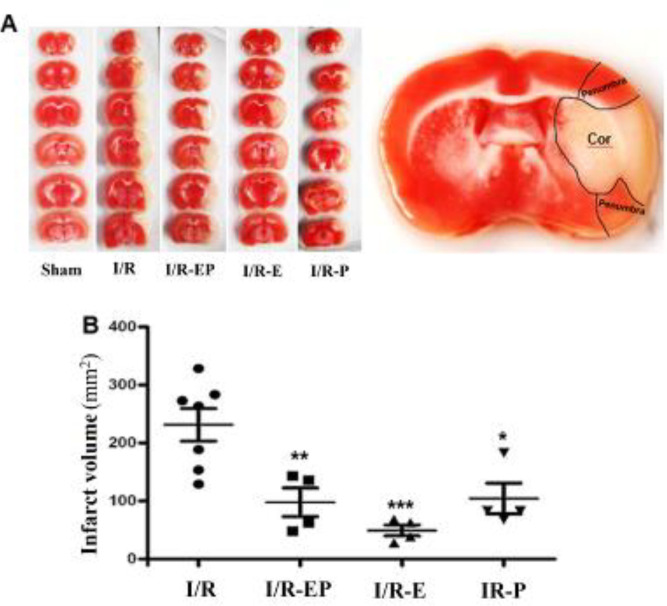
Infarct size was showed by TTC staining of brain sections. The white zone is infarct tissue and the red area is normal (A). Quantitative analysis of infarct volumes in the cerebral cortex showed that hormone (E2P, E2, and P) treatment significantly decreased infarct volume. (B) (*P≤0.05, **P≤0.01, and ***P≤0.001). TTC: triphenyl tetrazolium chloride; Cx: cerebral cortex; BG: basal ganglia

Infarct sizes were examined using vital staining with TTC 24 hr after stroke. Non-stained pale tissue was regarded as the cerebral infarct region, whereas intact vital tissue stained red. There was no detectable infarction in the sham-operated group, while extensive *infarction *was seen in the basal ganglia and cerebral cortex of I/R animals (Figure 2A). All hormone-treated groups including E2/P: 97.7 mm³ (*P*≤0.01(, E2: 49.3 mm³ (*P*≤0.001), and P: 104.2 mm³ (*P*≤0.05) showed a significant decline in the infarct volume compared with I/R group (231.4 mm³) (Figure 2B). 

***Glutamate transporters***

**Figure 3:**
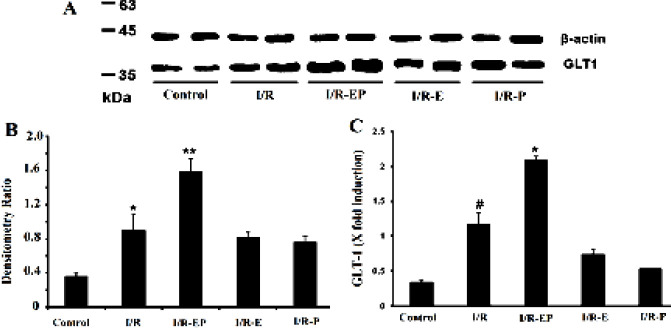
Gene and protein expression of EAAT3 after tMCAO and hormone therapy. EAAT3 positive cells shown by IHC in 3 groups and marked by arrows (A-C). These cells significantly increased in the I/R group compared with the control and also increased in the E2/P group compared with the I/R group (D). Also, EAAT3 gene expression increased after I/R and E2/P therapy significantly increased it compared with I/R (** P≤0.01, *P≤0.05)

EAAT3: excitatory amino acid transporter3; tMCAO: transient middle cerebral artery occlusion; IHC: immunohistochemistry; I/R: ischemia/reperfusion

We used RT-PCR, immunohistochemistry staining and Western blot analysis to investigate the effect of tMCAO on glutamate transporters (EAAT3 and GLT-1) expression in the penumbra region of ischemic rats and the possible effects of hormone therapy. Real-time PCR results showed a significant elevation of EAAT3 mRNA levels (*P*≤0.05) and increased numbers of EAAT3- positive cells after tMCAO (*P*≤0.05). This effect was enhanced after E2/P hormone administration and the numbers of EAAT3- positive cells were increased in the E2/P group compared with I/R (*P*≤0.01). In agreement with RT-PCR results, immunohistochemistry data revealed that the number of EAAT3 positive neurons and the intensity of protein on cell membranes were increased significantly in the cortical penumbra region of I/R group compared with controls. The number of intense EAAT3 positive neurons was enhanced again after a combination dose of EP treatment compared with I/R (*P*≤0.01). The number of EAAT3 positive cells was normalized and given as cell/mm^2^ (Figure 3).

**Figure 4:**
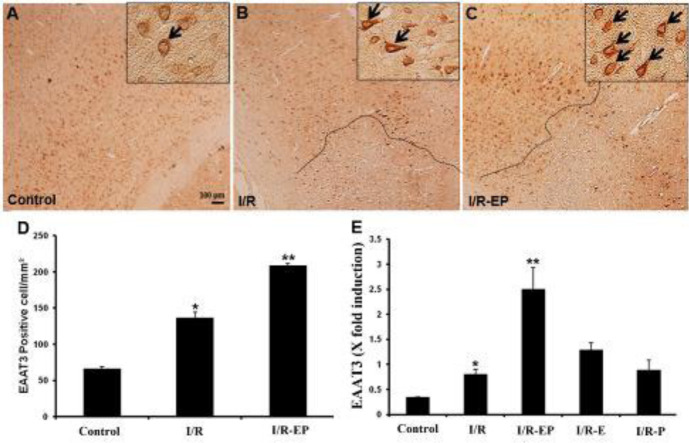
Gene and protein expression of GLT-1 protein after tMCAO and hormone therapy. Western blot results analysis showed that the protein levels of GLT-1 in the I/R group increased compared with control (*P≤0.05), and E2/P treatment significantly increased GLT-1 expression compared with I/R (**P≤0.01). Also, GLT-1 gene expression significantly increased after I/R (#*P*≤0.05), and E2/P therapy significantly increased it compared with I/R (*P≤0.05)

As shown in Figure 4, GLT-1 protein and mRNA expression were significantly increased after 1 hr ischemia followed by 24 hr reperfusion compared with controls (*P*≤0.05). Also, GLT-1 mRNA and protein were significantly higher in the E2/P group compared with I/R group (*P*≤0.05) 

## Discussion

In the present study, we investigated the changes in GLT-1 and EAAT3 mRNA and protein expression following transient focal cerebral ischemia and the possible neuroprotective role of steroids through regulating and modulating ischemia-induced glutamate transporter levels. Ischemia causes a complex cascade of physiological and pathophysiological events including oxidative stress, glutamate-induced excitotoxicity, apoptosis, and necrosis (5). In ischemic stroke, the neurotransmitter glutamate is widely released into the extracellular space (13). EAATs localized on the membrane of neurons and astrocytes effectively remove glutamate from the extracellular space. Thereby, they form the physiological platform of repetitive glutamate synaptic transmission and prevent an extracellular rising which could induce neuronal death (38, 39). It has been demonstrated that modulation of the glutamate transporters by the administration of ceftriaxone or N-acetylcysteine for 5 days before cerebral ischemia, induced brain tolerance to ischemia by significantly limiting stroke-related damage and normalizing Glu concentration (40). *In vivo* and *in* *vitro* studies have further shown that E2 and P have significant neuroprotective properties in several models of neurodegenerative diseases and under acute neuronal stress (41)*. *In particular, these steroids reveal their neuroprotective potency after traumatic injury, ischemia, and excitotoxicity (42, 43). Despite this evidence, the exact mechanisms which transmit neuroprotection on a tissue- and cellular level are not fully understood and probably manifold. It seems quite safe to conclude that regulation of excitotoxicity is one important aspect of this scenario. Consistent with earlier results, we have now shown that E2 and P individually and in combination can reduce the infarct volume and improve the functional deficits after transient focal cerebral ischemia (27, 29, 44, 45). In addition, we studied changes in gene and protein expression of GLT-1 and EAAT3 transporters after tMCAO, and the possible role of E2 and P. GLT-1 is mainly expressed by astroglial cells and plays an essential role in removing extracellular glutamate (15, 46). In our study, tMCAO significantly increased mRNA and protein level of GLT-1. Our results are in agreement with findings from Arranz *et al.* who observed a rapid up-regulation of GLT-1 in the subcortical white matter following transient MCAO. In addition, releasing glutamate from damaged axons whose cell bodies are located in the affected area can also increase extracellular glutamate and activate EAAT expression (47). Another study demonstrated a progressive increase in GLT-1 protein after global ischemia (48). We here found that E2/P treatment after ischemia significantly elevated GLT-1 mRNA and protein levels compared with the tMCAO group. In developmental studies, it has been shown that estradiol increases the expression and function of GLT-1 glutamate transporters in neonatal midbrain astrocytes. These hormones may protect neurons from excitotoxicity by inducing the release of protective factors from astrocytes, such as GDNF (Glial cell-derived neurotrophic factor (18, 49). EAAT3 is distributed in neuronal membranes and described as a postsynaptic glutamate carrier (14). We performed immunohistochemical studies to assess the localization of EAAT3 within the ischemic penumbra and showed that EAAT3 was localized at a high density on neuronal plasma membranes (Figure 3). This observation is in line with a previous report by Pradillo *et al.* who presented evidence of increased expression of this transporter after cerebral ischemia (39). We also observed that after tMCAO, EAAT3 protein and mRNA expression were significantly increased compared with the control group. Such an effect has also been described in hippocampal CA1 neurons after tMCAO (50), although expression patterns of EAATs also appear to vary after ischemia (50, 51). The activation of glutamate transporters can be triggered via extracellular glutamate concentrations which are typically elevated after MCAO (52). Previous studies have reported that an up-regulation of EAAT expression correlates with neuroprotection (53, 54). We assume that the observed up-regulation of GLT-1 and EAAT3 glutamate transporters after ischemia represents a self-compensative mechanism in order to modulate extracellular glutamate and attenuate glutamate excitotoxicity. Furthermore, we revealed for the first time that mRNA and protein expression of EAAT3 is further stimulated after E2/P treatment to reinforce this mechanism and improve neuroprotection even more. Additionally, these transporters could also absorb cysteine from the synaptic space. Thus, E2 can enhance the expression of GLAST and GLT-1 glutamate transporters by lowering extracellular cysteine levels along with glutamate (55-57). After transporting the amino acid cysteine into neurons and astrocytes, it is transformed into its monomeric form which is one of the most important precursors of glutathione (9, 58, 59). Glutathione can act as an important antioxidant reagent in the brain; therefore, the disturbance of its synthesis plays a vital role in several neurodegenerative diseases including cerebral ischemia (60, 61). Cysteine transport mechanisms together with glutamate are necessary for glutathione synthesis in CNS (62). Our results propose that E2/P treatment protects the brain from injury after ischemia due to their effects on glutamate and cysteine re-uptake which can reduce glutamate-induced neurotoxicity. 

GLT-1, glutamate transporter-1; tMCAO: transient middle cerebral artery occlusion; I/R: ischemia/reperfusion

## Conclusion

Our findings show that a combined E2/P treatment after ischemia protects brain tissue against glutamate neurotoxicity most likely via modulating glutamate transporter expression which in consequence induces glutamate re-uptake from extracellular space and prevents neurotoxicity. 
